# Validation Protocol of Vitamin D Supplementation in Patients with HIV-Infection

**DOI:** 10.1155/2016/5120831

**Published:** 2016-09-06

**Authors:** Elisabet Lerma-Chippirraz, Roberto Güerri-Fernández, Judit Villar García, Alicia González Mena, Ana Guelar Grinberg, María Milagro Montero, Luisa Sorli, Sonia Calzado, Juan Pablo Horcajada, Adolfo Díez-Pérez, Hernando Knobel Freud

**Affiliations:** ^1^Hospital del Mar, Infectious Diseases, Barcelona, Spain; ^2^Departament de Medicina, Universitat Autònoma de Barcelona, Barcelona, Spain; ^3^Hospital del Mar, Internal Medicine, Barcelona, Spain

## Abstract

Hypovitaminosis D and secondary hyperparathyroidism are frequent among HIV-infected patients. As there are no data about the best supplementation therapy both in treatment and in maintenance, we conducted an observational study of 300 HIV-infected patients for whom vitamin D and parathormone (PTH) had been measured in order to validate a protocol of vitamin D supplementation in patients with HIV-infection. Patients with vitamin D deficiency (defined as 25(OH)D < 10 ng/mL), insufficiency (defined as 25(OH)D < 20 ng/mL), or hyperparathyroidism (PTH > 65 pg/mL) were supplemented with cholecalciferol 16.000IU (0.266 mg) weekly (if deficiency) or fortnightly (if insufficiency or high PTH levels). Rates of normalization of 25(OH)D (levels above 20 ng/mL) and PTH levels (<65 pg/mL) were analyzed. Multivariate analysis of factors related to normalization was carried out. With a median follow-up of 2 years, 82.1% of patients with deficiency and 83.9% of cases with insufficiency reached levels above 20 ng/mL. However, only 67.2% of individuals with hyperparathyroidism at baseline reached target levels (<65 pg/mL). Independent factors for not achieving PTH objective were tenofovir (TDF) and protease inhibitors use. In HIV-infected patients with hypovitaminosis, the protocol of cholecalciferol supplementation normalized vitamin D levels regardless of antiretroviral regimen in a high proportion of patients but it was less effective to correct hyperparathyroidism.

## 1. Introduction

In recent years, a growing number of studies have reported high prevalence rates of vitamin D deficiency among HIV-infected patients [1]. The prevalence of hypovitaminosis goes from 42 to 95% in different studies [2–4]. In a previous study conducted in our hospital hypovitaminosis was reported in as many as 71.2% of HIV-infected patients (39.6% of them were vitamin D deficient as defined by levels below 10 ng/mL) [5].

The relevance of the high prevalence of hypovitaminosis D among HIV patients is that vitamin D is not only a well-established factor for bone disease [6–11], but also associated with nonskeletal conditions, including cardiovascular, immune regulation, cancer, and neurocognitive disorders [12–14]. Moreover, some studies suggest that severe vitamin D deficiency is associated with HIV progression, mortality, and AIDS events [2].

Risk factors for hypovitaminosis D are female sex, dark skin pigmentation, low dietary intake, seasonality, insufficient sun exposure, and high body mass index [15–19]. Recently we also identified as predictors of vitamin D deficiency in HIV-infected patients the existence of psychiatric comorbidity while lipoatrophy was a protective factor [5]. In addition to all classic risk factors, these subjects are also exposed to HIV-related factors associated with vitamin D deficiency such as immune activation, chronic inflammation, and viral and antiretroviral treatment with potential interactions on the vitamin D metabolism [20–26].

The systematic screening for vitamin D deficiency is controversial. The most recent EACS guidelines [27] suggest screening for hypovitaminosis D for every HIV-positive subject having a bone disease (low bone mineral density, previous fractures, or high risk for fractures) or other known factors for vitamin D deficiency. Vitamin D repletion is recommended when 25(OH)D levels are below 10 ng/mL. Furthermore it may be indicated in presence of vitamin D values between 10 and 30 ng/mL if associated with bone disease or secondary hyperparathyroidism. Elevated levels of PTH as a consequence of chronic vitamin D deficiency have been linked to bone loss [28]. Accordingly, EACS guidelines recommend vitamin D supplementation to reach levels above 20 ng/mL and normalizing serum PTH levels [27].

Arguments against the widespread screening for vitamin D deficiency in HIV-infected patients include the unclear benefit of vitamin D replacement for nonmusculoskeletal outcomes, the cost of laboratory testing and treatment, and the potential toxicities of some supplementation approaches [29]. Furthermore, the optimal repletion and maintenance dosing regimens remain to be established as well as the impact of vitamin D supplementation in preventing comorbidities [1]. Another study proposed that a dose of 16.000 IU calcidiol monthly during 9 months [30] was efficacy in decreasing the prevalence of hypovitaminosis D and the rates of associated hyperparathyroidism.

Therefore, accepting the evidence that adequate vitamin D levels are beneficial for HIV patients we implemented a protocol for vitamin D supplementation and analyzed the factors influencing the achievement of these target levels in a clinical population where a protocol for vitamin D supplementation has been implemented. Moreover, we also analyzed the relationship between vitamin D and PTH levels.

## 2. Materials and Methods

### 2.1. Patients and Study Design

An observational study was conducted at the Department of Infectious Diseases at Hospital del Mar (Barcelona, Spain), analyzing 300 HIV-infected out-patients who were naïve or on stable HAART (highly active antiretroviral therapy) for whom vitamin D levels had been measured (fasting status). Patients were followed up in our department and visited between June 2010 and October 2013. The study was approved by the hospital ethical committee and all subjects provided written informed consent. All patients' data were anonymized for data management and statistical analysis. As an observational study, there was no control arm for comparison and hence no randomization.

### 2.2. Quantification of Laboratory Values

Vitamin D (competitive electrochemiluminescence protein binding assay, Cobas e602.Roche Diagnostics, Germany) status was categorized as insufficient when <20 ng/mL and deficient when <10 ng/mL and hyperparathyroidism as PTH levels (solid-phase, two-site chemiluminescent enzyme-labeled immunometric assay; IMMULITE 2000, Siemens; Los Angeles, CA, USA) >65 pg/mL. Secondary laboratory variables included serum levels of calcium (8.5–10.5 mg/dL), phosphate (2.5–4.8 mg/dL), alkaline phosphatase (40–129 IU/L), CD4 and CD8 lymphocytes counts, and HIV viral load (COBAS, AmpliPrep/TaqMan HIV-1 test, Roche Diagnostics).

The following demographic and clinical data were assessed: gender, age, race, risk factors for HIV-infection, HAART, and hepatitis C and B coinfection. For the analysis were excluded patients with renal insufficiency (defined as glomerular rate filtration <60 mL/min, estimated using Modification of Diet in Renal Disease (MDRD) equation), hepatic insufficiency (defined as Child Pugh stage C), patients under chemotherapy, and those already receiving vitamin D, bisphosphonates, and calcium supplements (and the other classical exclusion criteria).

**Figure 1:**
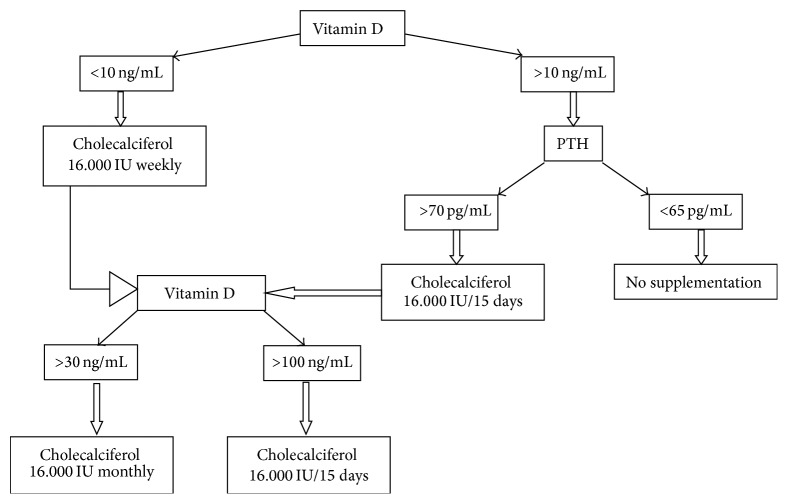
Protocol of vitamin D supplementation.

Subjects were supplemented with 16.000 IU or 0.266 mg of vitamin D3 (cholecalciferol, Hidroferol®, FAES FARMA) weekly, fortnightly, or monthly according to vitamin D baseline and PTH concentrations and according to the supplementation protocol designed by the Department of Infectious Diseases at Hospital del Mar (Figure 1). Those patients with vitamin D below 10 ng/mL were supplemented with 16.000 IU of cholecalciferol weekly. Patients with vitamin D above 10 ng/mL were replaced according to PTH values, though for those with PTH > 70 ng/mL 1 dose fortnightly was used and those with PTH < 65 ng/mL did not receive supplements. All subjects were reevaluated after 4–6 months and at every outpatient visit thereafter up to two years of follow-up. If vitamin D levels reached levels between 30 ng/mL and 100 ng/mL, the dose was modified to 16.000 IU fortnightly. When serum vitamin D reached levels above 100 ng/mL the dosage was modified to 16.000 IU every month. During the follow-up all patients were reevaluated 3 times. Subjects with normal vitamin D levels were not supplemented. Vitamin D toxicity was defined as concentrations above 150 ng/mL [31].

### 2.3. Statistical Analysis

Univariate analyses were performed with chi-square test and ORs and its 95% confidence interval was estimated by logistic regression.

Data of 300 participants from the 3 follow-up moments were pooled resulting in 798 registers. With the data arranged in this manner a Linear Mixed Model was performed to analyse the relationship between PTH and vitamin D, adjusted by TDF. In this model the individual (the identifier) was included as a random effect.

To analyse the factors associated to achieve the objective of 25(OH)D level above 20 ng/mL and PTH level below 65 pg/mL a chi-square analysis was done for the univariate analysis and a logistic regression model for the multivariate analysis; both analyses were done in patients with baseline 25(OH)D < 20 ng/mL and baseline 25(OH)D < 10 ng/mL.

Statistical analysis was performed with R Statistical Package (R Foundation for Statistical Computing, Vienna, Austria; Version 2.15.1).

## 3. Results

### 3.1. Study Population

The characteristics of the 300 patients included in the study are presented in Table 1. The median age of participants was 46 years (range 41–52), 219 (73%) were males, and 253 (84.3%) were caucasians. Predominant risk groups for HIV-transmission were IDU (intravenous drug users) (36.7%) and MSM (men having sex with men) (35.3%). At the time of sampling 283 patients (95.3%) were on cART; from those 173 (57.7%) were exposed to TDF, 130 (43.3%) to nonnucleoside reverse transcriptase inhibitors (NNRTI), and 173 (55%) to ritonavir-boosted protease inhibitor regimen (PI). The proportions of vitamin D deficiency and insufficiency were 28% and 62%, respectively, and 45.7% of the patients had secondary hyperparathyroidism.

The protocol described in Figure 1 was intended to be applied in the 300 HIV-infected patients.

### 3.2. Changes in Vitamin D and PTH

At baseline, 84 patients were vitamin D deficient and 186 insufficient. All patients, both deficient and insufficient, were offered supplementation but all those who declined this option were not supplemented. A total amount of 22 patients of those with vitamin D deficiency and 58 of those with insufficiency declined supplementation.

62 patients with vitamin D deficiency (73.8%) were supplemented (Table 2). After completing 24 months, 2 (3.2%) participants receiving vitamin D had a trough serum 25(OH)D concentration <20 ng/mL compared to 13 (59.1%) of those not supplemented (*p* < 0.0001). Vitamin D objective was influenced by neither TDF exposure (*p*: 0.65) nor nonnucleoside exposure (*p*: 0.23). A high proportion of patients on PI therapy achieved 25(OH)D concentration >20 ng/mL (89.4% versus 73%; *p*: 0.08); however, the difference did not reach statistical significance. At the multivariate analysis, the only significant factor associated with achieving the objective of 25(OH)D concentration >20 ng/mL was cholecalciferol supplementation (*p* < 0.0001).

Table 3 shows the factors associated with PTH objective (PTH < 65 pg/mL) in patients with vitamin D deficiency. Vitamin D supplements had no effect over PTH objective, but at the logistic regression tenofovir exposure was a risk factor associated with not achieving PTH objective (OR: 3.77 (1.36–10.43)).

The results were similar in those subjects with vitamin D insufficiency. The proportion of participants with vitamin D <20 ng/mL was 62% (186 patients) of whom 128 subjects were supplemented with cholecalciferol. More patients in the supplemented regimen achieved vitamin D concentration >20 ng/mL after treatment than those not supplemented (95.5% compared with 53.8%, resp.; *p* = 0.0001).  Table 4 summarizes the factors associated with reaching both objectives, vitamin D and PTH. As in the patients with vitamin D deficiency, PI based therapies and those with TDF exposure were associated with a higher risk of not achieving PTH levels under 65 pg/mL.

Although cholecalciferol supplementation did not seem to correct the secondary hyperparathyroidism in the qualitative analysis it had an effect over PTH concentration so for each increasing unit of vitamin D, PTH decreased 0.213 pg/mL (*p* < 0.001). Moreover, PTH values were significantly higher, 10.643 pg/mL, in those patients exposed to TDF (*p* < 0.001) (Table 5).

No changes on HAART due to vitamin D for PTH levels were made. Those HAART switches during supplementation were made for either virologic failure or adverse events related to antiretroviral therapy.

There were no differences between patients supplemented and those not with respect to the frequencies of possible adverse events including infectious diseases, fractures, cancer, and cardiovascular diseases, so we considered there were no events attributable to vitamin D replacement. During the course of the study no patient in replacement group presented toxicities (no hypercalcemia). The mean viral load, CD4 number and CD4%, calcium, phosphate, and alkaline phosphatase measurements did not differ between baseline and the end of the study.

## 4. Discussion

Considering that hypovitaminosis is associated with unfavorable outcomes of some chronic infections such as HIV [32] and the increasing evidence of its role in innate and cell mediated immunity [33] we proposed a practical scheme for vitamin D supplementation. When designing this protocol we focused on several problems. First of all, the recent development of the once-daily single tablet regimen has optimized HAART regimens and improved long term adherence, as well as quality of life [34]. But HAART has also increased life expectancy and therefore age-related morbidities as well as the need for concomitant medication. In a recent study, suboptimal adherence was associated with greater pill burden and higher maximum number of doses per day [35]. The aim of the proposed scheme was the avoidance of adherence problems; thus we designed a protocol in which we could give the least possible number of doses: maximum 1 per week and at least 1 monthly. On the other hand recommendations on screening and vitamin D supplementation remain sparse and controversial and the target level of vitamin D to be reached after replacement is not yet known. Some investigators consider that a minimum serum concentration of 30 ng/mL is necessary, whereas others suggest that the calciotropic functions of vitamin D are enhanced with higher concentrations of serum 25(OH)D within the current reference range [36–39].

The results of this study demonstrate that supplementation of HIV-infected individuals with vitamin D is well tolerated, safe, and effective, as more than 95% of the patients achieved vitamin D levels above 20 ng/mL, with the proposed schedule of administration.

In the current study, as in others, supplementation did not significantly affect calcium, phosphorus, and alkaline phosphatase during the period of observation. In addition, no differences were observed in the mean viral load, CD4 number, and CD4% between baseline and the end of the study. Moreover, in contrast to other studies, neither baseline concentration of vitamin D nor vitamin objectives were influenced by the cART use [20, 22, 40].

On the other hand, PTH elevation plays a direct role over bone disease among HIV-infected patients, as it acts on bone to release calcium and persistent PTH elevations are associated with bone loss across a range of clinical conditions [41–43]. Studies on evaluation of secondary hyperparathyroidism in HIV-infected patients are scarce; as a result there are few data on the impact of this condition on the health of individuals [44].

Because low vitamin D was associated with PTH increases, we hypothesized that vitamin D repletion may reduce the risk of PTH abnormalities. Many studies have shown that serum PTH is inversely related to serum 25(OH)D, and that with the increase in serum 25(OH)D there is a decrease in serum PTH that usually reaches a plateau. In the Endocrine Society guidelines [45], the statement is made that serum PTH reaches a plateau at a threshold of 30 ng/mL, but this is supported by only a few references, since in recent literature there is a large variability in the reported level of serum vitamin D at which PTH reached a plateau or was maximally suppressed [46]. In our study, as in others before, serum PTH was inversely correlated with serum 25(OH)D but no threshold as defined by suppression of serum PTH was found. Sai et al. showed similar results, but they do found a threshold for bone markers that increased only below a vitamin D of approximately 18 ng/mL, so they conclude that vitamin D insufficiency should be defined as 25(OH)D <20 ng/mL as it relates to bone. Although there is not much literature about PTH normalization, we report that 66.7% of the patients achieve PTH levels <65 pg/mL after 2-year follow-up. We hypothesized that a longer follow-up may increase the number of patients that correct hyperparathyroidism because after supplementation the time it takes to normalize PTH is not set.

In different studies vitamin D has been associated with TDF-linked hyperparathyroidism emphasizing that both, TDF use and vitamin D status, influence in PTH values. Although the mechanism by which TDF produces hyperparathyroidism is unclear, a previous study suggests that the increased hydroxylation rates and tubular phosphate losses, which drive calcium preservation and possibly altered bone metabolism, are dependent on vitamin D status [47]. Moreover PTH elevations have been observed in patients taking both NNRT and PI [48–50]. We observed that patients under TDF treatment presented PTH values significantly higher which is consistent with other studies [51]. Although vitamin D treatment may decrease PTH in persons taking TDF in the absence of measurable vitamin D deficiency, in our study TDF or IP exposure was a risk factor for not achieving PTH objective. So it is suggested that because 1,25(OH)D directly decreases PTH and vitamin D treatment increases 1,25(OH)D and its direct effect may decrease PTH. But as hyperparathyroidism may be multifactorial in HIV-infected patients and TDF may have effects on other factors that directly increase PTH, vitamin D supplementation could be not enough to normalize PTH but to improve the levels as we have seen in the present study.

Limitations of the present study are mainly related to the observational design nature of the current analyses. In this respect, the results reported herein are only associations from which no conclusions regarding causality can be drawn. The 25(OH)D measurements were done along the whole year, independently of seasonality. On the other hand we did not examine sun exposure, nor intakes of dietary calcium, although dietary calcium deficiency is rare in our population due to Mediterranean diet, and serum calcium and phosphorus were normal during the complete follow-up. Calcium urinary excretion was not measured.

In conclusion we demonstrate that the proposed scheme for vitamin D supplementation in HIV-infected patients is safe and valid for correcting vitamin D abnormalities and to improve raised PTH levels, but not enough for normalizing them, especially in patients exposed to tenofovir or protease inhibitors.

## Figures and Tables

**Table 1:** Demographic and clinical characteristics of the study population.

Age (years)	Mean (SD): 46.96 (9.61)
	Median range: 46 (41–52)

Sex *n* (%)	Male: 219 (73)
	Female: 81 (27)

Ethnic background *n* (%)	White: 253 (84.3)
	Hispanic: 27 (9)
	Black: 9 (3)

Risk group *n* (%)	IDU: 110 (36.7)
	MSM: 106 (35.3)
	Heterosexual: 74 (24.7)
	Others: 7 (3.3)

HAART *n* (%)	Yes: 283 (95.3)
	No: 17 (4.7)
	NNRTI: 130 (43.3)
	PI: 165 (55)
	TDF: 173 (57.5)
	ABC: 63 (21)

Serum 25(OH)D category *n* (%)	<10 ng/mL: 84 (28)
	<20 ng/mL: 186 (62)
	>20 ng/mL: 114 (38)

PTH > 65 pg/mL *n* (%)	Yes: 137 (45.7)
	No: 163 (54.3)

**Table 2:** Patients with vitamin D < 10 ng/mL at baseline. Objective vitamin D > 20 ng/mL. Univariate and multivariate analysis.

	Univariate analysis			Multivariate analysis	
	Objective yes: 69 (82.1%) *n* (%)	Objective no: 15 (17.9%) *n* (%)	*p*	OR (95% CI)	*p*
*VitD supplements*					
Yes	60 (96.8)	2 (3.2)	0.0001	66.76 (10.06–442.85)	0.0001
No	9 (40.9)	13 (59.1)			

*TDF exposure*				0.33 (0.05–1.92)	0.22
Yes	32 (84.2)	6 (15.8)	0.65		
No	37 (80.4)	9 (19.6)			

*NNRTI exposure*				1.12 (0.69–18.38)	0.93
Yes	26 (76.5)	8 (23.5)	0.26		
No	43 (86)	7 (14)			

*PIs exposure*				0.19 (0.01–2.97)	0.23
Yes	42 (89.4)	5 (10.6)	0.08		
No	27 (73)	10 (27)			

**Table 3:** Linear Mixed Model, PTH dependent variable.

Slope 95% CI					
Variable	Slope	Lower	Upper	Correlation	*p* value
VitD	−0.213	−0.291	−0.134	−0.592	<0.001
TDF	10.643	4.861	16.425	—	<0.001

**Table 4:** Patients with vitamin D < 10 ng/mL at baseline. Objective PTH < 65 pg/mL.

	Univariate analysis			Multivariate analysis	
	Objective yes 56 (66.7)	OR (95% CI)	*p*	OR (95% CI)	*p*
*VitD supplements*					
Yes	41 (66.1)	0.91 (0.32–2.85)	0.86	1.05 (0.34–3.24)	0.92
No	15 (68.2)				

*TDF exposure*					
Yes	20 (52.6)	3.24 (1.26–8.35)	0.01	3.77 (1.36–10.43)	0.01
No	36 (78.3)				

*NNRTI exposure*					
Yes	23 (67.3)	1.08 (0.43–2.72)	0.87	3.36 (0.55–20.50)	0.19
No	33 (66)				

*PIs exposure*					
Yes	28 (59.6)	2.11 (0.82–5.46)	0.12	7.04 (1.09–45.47)	0.04
No	28 (75.7)				

**Table 5:** Analysis patients with vitamin D < 20 ng/mL at baseline.

	Objective VitD > 20 ng/mL Yes: 156 (83.9) *n* (%)	*p*	Objective: PTH < 65 pg/mL	*p*
*VitD supplements *				
Yes	128 (95.5)	0.0001	90 (67.2)	0.43
No	28 (53.8)		39 (73.1)	

*TDF exposure*				
Yes	80 (89.9)	0.03	54 (60.7)	0.02
No	76 (78.4)		74 (76.3)	

*NNRTI exposure*				
Yes	75 (85.5)	0.58	61 (73.5)	0.22
No	76 (82.5)		67 (65)	

*PIs exposure*				
Yes	83 (84.7)	0.75	61 (62.2)	0.04
No	73 (83)		67 (76.1)	
